# Mapping Recombination Landscape and Basidial Spore Number in the Button Mushroom *Agaricus bisporus*

**DOI:** 10.3389/ffunb.2021.711330

**Published:** 2021-08-20

**Authors:** Anton S. M. Sonnenberg, Narges Sedaghat-Telgerd, Brian Lavrijssen, Patrick M. Hendrickx, Karin Scholtmeijer, Johan J. P. Baars, Richard G. F. Visser, Arend van Peer

**Affiliations:** Plant Breeding, Wageningen University and Research, Wageningen, Netherlands

**Keywords:** meiotic recombination, recombination landscape, basidial spore number, QTL analysis, breeding strategy

## Abstract

The button mushroom *Agaricus bisporus* is represented mainly by two varieties, a secondarily homothallic variety with predominantly two heterokaryotic spores per basidia and a heterothallic variety with predominantly four homokaryotic spored basidium. Both varieties also differ in their recombination landscape with the former showing crossovers (CO) predominantly at chromosome ends whereas the latter has a more evenly distribution of CO over the chromosomes. The two varieties are compatible, and this has been used to study segregation of the basidial spore number (BSN) and the genomic positions of recombination, i.e., the CO landscape, in order to find the underlying genetic determinants. Knowledge on genes controlling CO positions might facilitate either the conservation of favorable allele combinations or the disruption of unwanted allele combinations to reduce linkage drag. For BSN, in total seven QTL were found with the major QTL on chromosome 1 explaining ca. 55% of the phenotypic variation. It appeared, however, difficult to map the recombination landscape. This phenotype can only be assessed in the meiotic offspring of an intervarietal hybrid which is a laborious and difficult task. Nevertheless, this was done, and we were able to map three QTLs for this trait, two on chromosome 1 and one on chromosome 2 not overlapping with the QTL for BSN. The hurdles encountered are discussed and a new strategy is proposed that can solves these. We propose to use two genetically unrelated mapping populations both offspring of a cross between a var. *bisporus* and a var. *burnettii* homokaryon and thus segregating both for CO and BSN. Homokaryotic offspring of both populations can be intercrossed without limitation of mating incompatibility and marker homozygosity and the hybrid mushrooms directly used to map BSN. Homokaryotic offspring of these hybrid mushrooms can be genotypes to assess CO positions using next generation sequencing technologies that will solve marker problems encountered, especially for genotyping chromosome ends. This new approach can be a useful strategy for a more efficient breeding strategy for mushrooms in general.

## Introduction

To unravel the genetic base of different life cycles in basidiomycetes, the button mushroom, *Agaricus bisporus* is a useful model species since it represents three interfertile varieties with considerable differences in life cycles. *A. bisporus* var. *bisporus* is predominantly pseudohomothallic, var. *burnettii* predominantly heterothallic and var. *eurotetrasporus* true homothallic (Raper and Raper, 1972; Callac et al., 1993, 2003). Approximately 90% of the var. *bisporus* basidia bear two spores each receiving non-sister nuclei after meiosis (Kerrigan et al., 1993; Sonnenberg et al., 2016) and these produce fertile heterokaryons after germination. On rare four-spored basidia each spore receives one haploid nucleus and germinates into an infertile homokaryon, tri-spored basidia produce likely two homokaryotic and one heterokaryotic spores (Pelham, 1967; Elliott, 1972). The var. *burnettii* contrasts var. *bisporus* in forming predominantly four-spored basidia resulting in homokaryons after germination of spores and a minority of basidia bear two or three spores (Callac et al., 1993). Next to the difference in sexual reproduction, the main varieties *bisporus* and *burnettii* also differ in recombination landscape. Whereas in var. *burnettii* crossovers (CO) are found more or less evenly spread over chromosomes, CO in var. *bisporus* are predominantly found at the extreme ends of chromosomes (Foulongne-Oriol et al., 2010; Sonnenberg et al., 2016, 2020).

The present commercial cultivars of the button mushroom are represented by var. *bisporus*. The low percentage of homokaryotic offspring, needed for outbreeding, and the CO restriction to chromosome ends, resulting in linkage drag, severely hamper breeding of this important mushroom crop (Sonnenberg et al., 2017). Introduction of the genetic components underlying the heterothallic life cycle with four-spored basidia and the normal CO distribution of var. *burnettii* into var. *bisporus* would facilitate breeding of the species enormously. In addition, identification of genes involved in CO positioning will be of interest for mushroom breeding in general, including breeding of other organisms. Control of CO position in breeding programs will either allow the conservation of favorable allele combinations or the disruption of unwanted allele combinations to reduce linkage drag. However, the genetic bases for the number of spores per basidia and for the positioning of CO are unknown. Many genes involved in meiosis are conserved, but especially genes for structural components of the recombination machinery do not have orthologs outside their taxa (Grishaeva and Bogdanov, 2018) and sometimes do not even have homologs within the same genus (Young et al., 2004). In addition, no genes involved in positioning of CO at the extreme ends of chromosomes have been identified yet.

The compatibility of var. *bisporus* and var. *burnettii* offers a unique possibility to study the genetic base of the CO distribution and of basidial spore number (BSN) by segregation analysis. To our knowledge, CO positioning in meiosis is a trait that has not been studied before in this way. To study this trait, one should be aware that it is not visible in the mapping population, which in mushroom breeding refers to homokaryotic spores derived from the F_1_ hybrid mushroom. In order to study the effect of the genotype of the mapping population on the CO landscape, these homokaryons must be crossed with compatible homokaryons to produce F_2_ mushrooms. These F_2_ mushrooms can be generated either by intercrossing the mapping population or outcrossing with suitable tester homokaryons, each with its own pros and cons. Subsequently, genotyping of a sufficient number of homokaryotic offspring of each of these F_2_ hybrid mushrooms must be done to reveal the effect of the genetic constitution of the selected individuals of the mapping population on CO positioning, which is a laborious task. Meanwhile, specific markers must be generated that allow the quantification of the CO landscapes and that are manageable in labor and costs. In this paper we describe a first approach to map quantitative trait loci (QTL) for basidial spore number and the CO landscape and the hurdles we encountered. We will elaborate on the lessons learned and outline a new strategy that could avoid most of these problems. The proposed approach will be an example for breeding of mushrooms for complex traits.

## Materials and Methods

**Figure 1:**
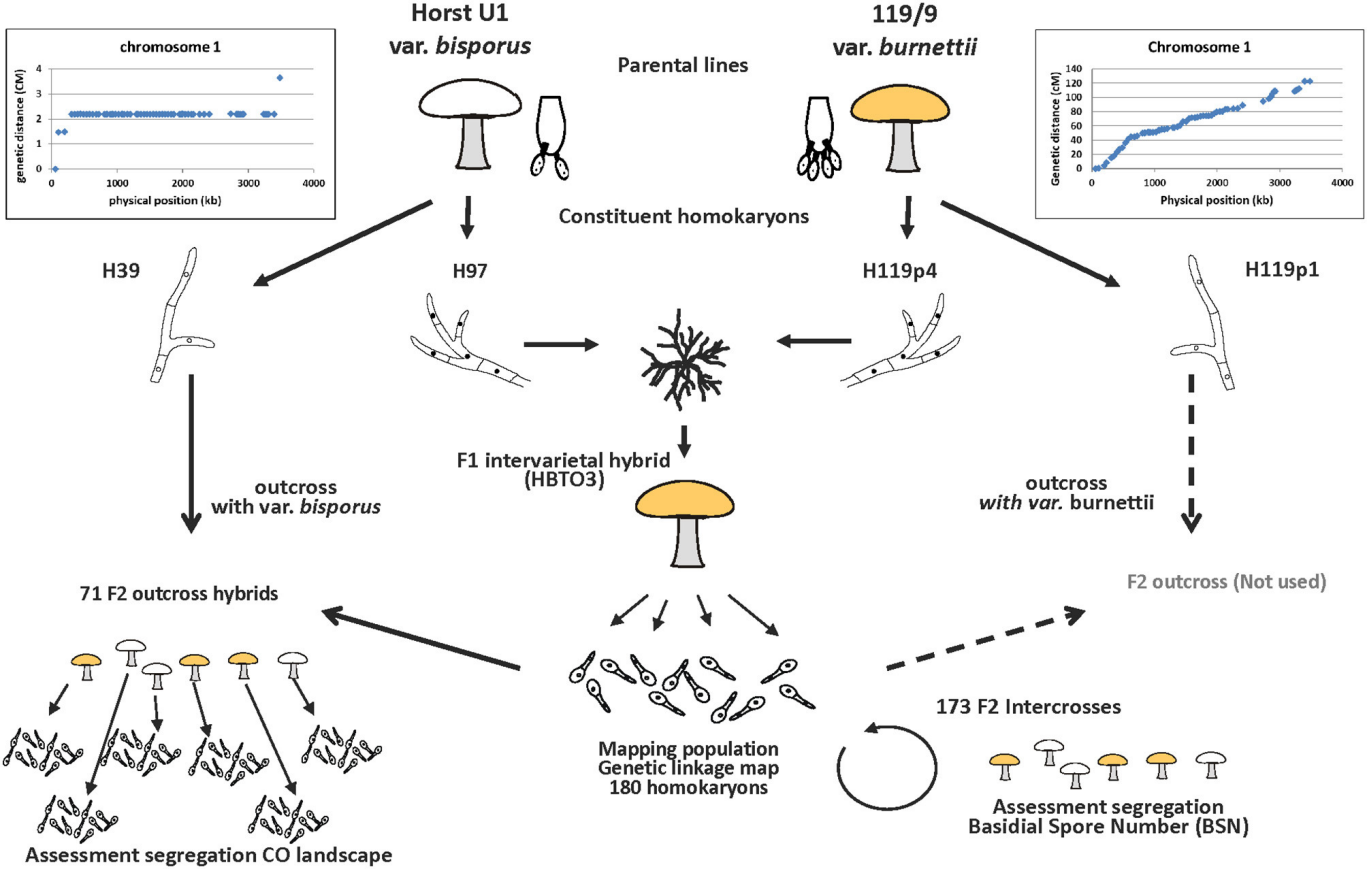
Experimental setup for mapping the basidial spore number and crossover (CO) landscape. The donors of the parental homokaryons are var. *bisporus* (predominantly 2-spored basidia and CO at chromosome ends) and var. *burnettii* (predominantly 4-spored basidia and CO evenly distributed over chromosomes). A constituent homokaryon of var. *bisporus* (derived from the commercial variety Horst U1) and a constituent homokaryon of the var. *burnettii* 119/9 (wild isolate) were crossed (F_1_ intervarietal hybrid HBT03) to generate a mapping population (haploid offspring of HBT03). A selection of this mapping population was subsequently intercrossed to map the basidial spore number (BSN) and outcrossed with the var *bisporus* homokaryon H39 to map the CO landscape.

An outline of the experimental setup is given in Figure 1. It depicts the origin of the parental homokaryons, the generation of the F_1_ and F_2_ mapping populations, the intercross to map the Basidial Spore Number (BSN) and the outcross to map the CO landscape.

### Genetic Linkage Map Based on the HBT03 Intervarietal Hybrid

To generate a mapping population, a homokaryon of var. *bisporus* and var. *burnettii* were crossed. For the former, one of the constituent homokaryons (H97) of the commercial variety Horst U1 was used and for the latter one of the constituent homokaryons (H119p4) of the wild var. *burnettii* variety 119/9 (Sonnenberg et al., 2020). The F_1_ intervarietal hybrid, designated as HBT03, was cultivated as described previously (Weijn et al., 2012) and a spore print of one of the mushrooms was spread in a dilution series on Malt Mycological Peptone agar medium (Sonnenberg et al., 1996). To avoid bias for selection of early/fast growing colonies, single spore isolates (SSIs) were picked up from the first day of visual germination (11 days after spreading spores) up to 39 days, illustrating the large variation in germination rate of the HBT03 intervarietal hybrid offspring. A total of 180 homokaryotic SSI were selected using a PCR marker linked to the mating type (Gao et al., 2013). Illumina reads of H119p4 generated previously (Sonnenberg et al., 2020) were mapped on the H97 genome to identify SNPs and a total of 508,912 SNPs was detected (16.5 SNPs/kb). The mapping population of 180 homokaryons were genotyped with a KASP assay (Kompetitive Allele-Specific PCR). For this, 215 SNP markers were selected that are evenly distributed over chromosomes. Based on the KASP genotyping data, a genetic linkage map was generated using R (version 3.6.3) in combination with the package ASMap (version 1.0-4) (Taylor and Butler, 2017). The genetic distances were calculated using Kosambi distance function.

### Mapping the Basidial Spore Number Loci

Homokaryons of the HBT03 derived mapping population were selected for mating to generate intercrosses (Figure 1). Three criteria were used for this selection: each homokaryon in a pair has an opposite mating type, each homokaryon has at least 13 COs (one per chromosome) as far as possible and both parental genomes of the F_1_ HBT03 (H97 and H119p4) should on average be equally represented in the total selection. Forty-two homokaryons were selected to generate 230 heterokaryotic F_2_ that were cultivated for fruiting. Of all inoculated hybrids, 173 did fruit and were examined for the number of spores to map the BSN loci. The parental lines (Horst U1 and 119/9) and the intervarietal hybrid (HBT03) were cultivated each on two trays. From each tray, one mushroom was selected of which three lamellae were examined for the number of spores per basidia (50 basidia per lamella). In total, 52,800 basidia were examined. The expected number of homokaryotic SSIs produced by the parental lines and the HBT03 were calculated according to Kerrigan et al. (1994), where 1- and 2-spored basidia have 0, 3-spored basidia have 2, and 4-spored basidia have four homokaryotic SSI. For QTL mapping, the average basidial spore number was calculated for each intercross. The genotypes of the intercrosses were reconstructed based on the genotypes of the 42 mated homokaryons of the mapping population. The genetic linkage map generated of HBT03 offspring as described above was used as input. The QTL analysis for BSN was performed using R (version 4.0.2) in combination with the package QTL2 (version 0.22-11) (Broman et al., 2018). Genotype probabilities were calculated using the Kosambi mapping function and the Haley–Knott Regression was used to identify significant QTLs.

### Comparison of CO Distribution in Parental Lines and in the HBT03 Intervarietal Hybrid

Three sets of data were used to compare the crossover distribution of the parental lines Horst U1 (var. *bisporus*), 119/9 (var. *burnettii*), and the HBT03 intervarietal hybrid. (A) for the parental type var. *bisporus*, data were used from resequenced homokaryotic and heterokaryotic (constituent nuclei recovered via protoplasting) offspring of the commercial variety Horst U1 (Sonnenberg et al., 2016), which allowed a precise positioning of CO along chromosomes. (B) for the parental type var. *burnettii* Genotyping by Sequencing data from offspring of strain 119/9 were used (Sonnenberg et al., 2020). Due to homozygosity of large parts of the genomes of the constituent nuclei of strain 119/9 (H119p1 and H119p4) only data from chromosomes 1, 3, 10 and 11 could be used. (C) for the HBT03 intervarietal hybrid, data from the genetic linkage map of HBT03 were used as described above. For the latter two sets of data, the physical midpoint of each marker pair was used to position COs. To compare all data, chromosomes were scaled to unit length by dividing the physical position of a CO by the length of the relevant chromosome. CO frequencies were subsequently plotted against their position on the standardized chromosome.

### Assessing Recombination Landscape in the Haploid Offspring of the F_2_ Outcross Hybrid Populations

A selection of the HBT03 mapping population was outcrossed with var. *bisporus* homokaryon H39, which shifts the CO position landscape more toward chromosome ends. To assess CO positions and to keep costs affordable, marker pairs were therefore chosen with one marker as far as possible toward the chromosome end and a second marker 150–200 kb inwards from the chromosome end. The latter markers function as the “border” between end-CO and middle-CO. CO between marker pairs left and between marker pairs right will thus be registered as CO at ends, and CO between the two middle markers as CO in the middle. Markers were designed to discriminate between the parents of the mapping population on the one hand (H97 and H119p4) and the tester homokaryon H39 on the other hand. For each of the F_2_ outcross hybrid, fractions of a CO at the ends (fractions 1 and 3) and in the middle (fraction 2) were calculated for each of the chromosomes in each of the individuals within the set of homokaryotic offspring of a hybrid. The number of homokaryotic individuals with a CO within a marker pair was divided by the number of individuals for which data were available for both markers in that pair to correct for differences in number of individuals in sets of homokaryotic offspring. The CO at ends and middle averaged over all chromosomes were calculated as follows: Ends Average equals the sum of CO at chromosome ends of all chromosomes divided by the total number of CO. The Middle Averages equals the sum of CO in the middle of all chromosomes divided by the total number of CO.

### QTL Mapping of the CO Landscape

The genotypes of the HBT03 mapping population, the calculated ends averages and middle averages of the offspring of the F_2_ outcross hybrids and the genetic map as described above were used as input for QTL analysis for CO landscape using R (version 4.0.2) in combination with the package QTL2 (version 0.22-11). Genotype probabilities were calculated using the Kosambi mapping function and the Haley-Knott Regression was used to identify significant QTLs.

## Results

### Linkage Map

**Figure 2:**
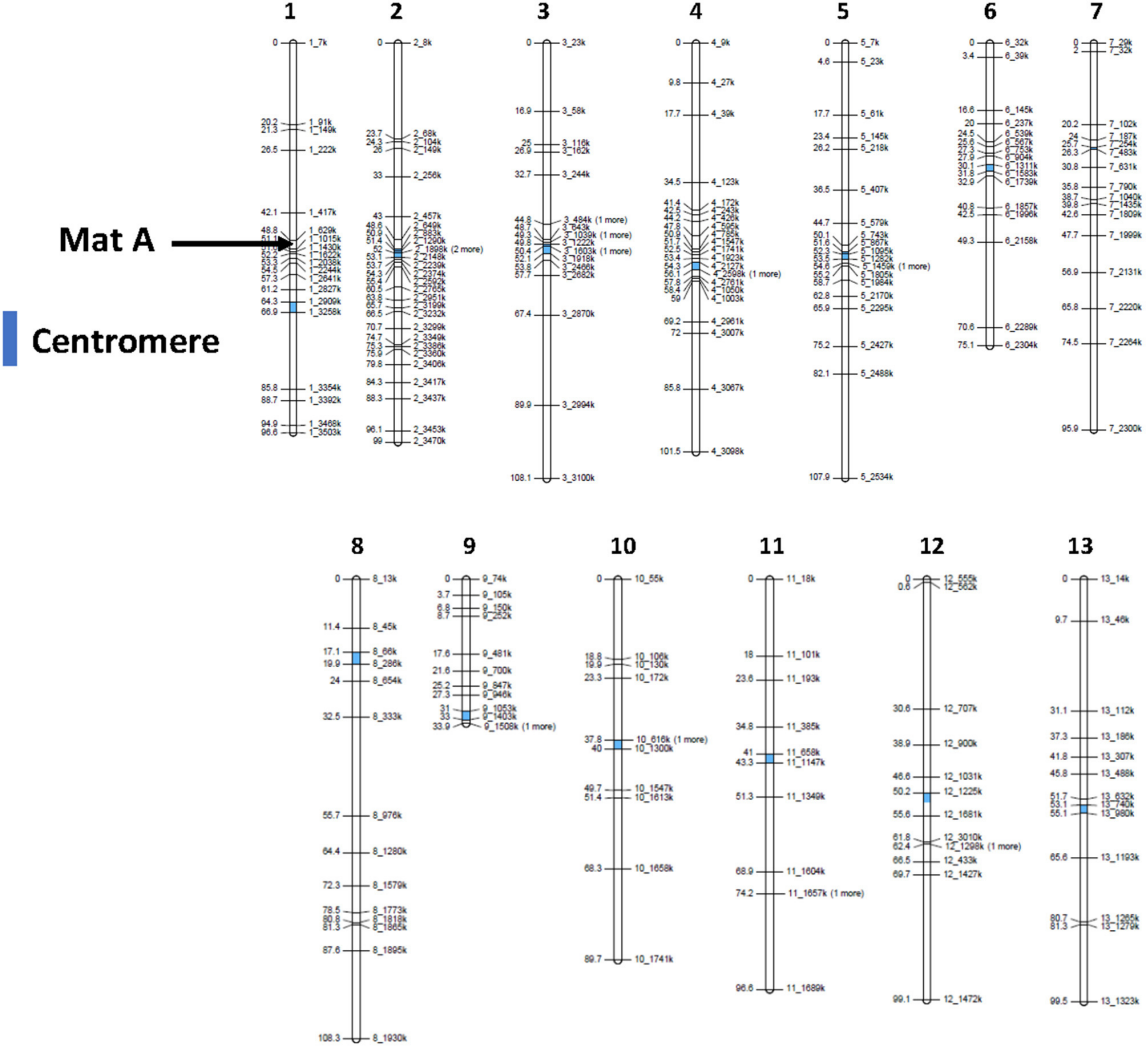
Linkage map generated from an offspring of a cross between H97 (homokaryon of a var. *bisporus*) and H119p4 (homokaryon of a var. *burnettii*). 180 Homokaryons were genotyped with 215 SNP markers. The position of centromeres (Sonnenberg et al., 2020) are indicated in blue. Map statistics are presented in Supplementary Table 1.

The mapping population was isolated as a homokaryotic offspring from a cross between var. *bisporus* (homokaryon H97) and var. *burnettii* (homokaryon H119p4), the former producing predominantly 2-spored basidia and CO restricted to chromosome ends, whereas the latter produces predominantly 4-spored basidia and shows a more even distribution of COs over chromosomes (Sonnenberg et al., 2016, 2020). A total of 180 homokaryotic SSIs were genotyped with 215 SNP markers, distributed well over the entire genome. The exception was the right arm of chromosome 9 where no SNP markers could be generated since this region harbors the ribosomal DNA cluster. The total genetic map length is 1,211 cM representing 13 linkage groups (LGs), corresponding to the number of chromosomes (Royer et al., 1992; Sonnenberg et al., 1996) (Figure 2, for map statistics see Supplementary Table 1). The map length is very similar to the previously published *bisp* x *burnettii* map (Foulongne-Oriol et al., 2010). Seventy-six markers deviate from the expected Mendelian ratio (1:1 ratio, chi-square test, *P* < 0.05). LG2 and especially LG3 are the main source of skewed segregating markers with an overrepresentation of the genome by H97 var. *bisporus* (for genotype of mapping population see Supplementary Figure 1).

### QTL Mapping of BSN

**Figure 3:**
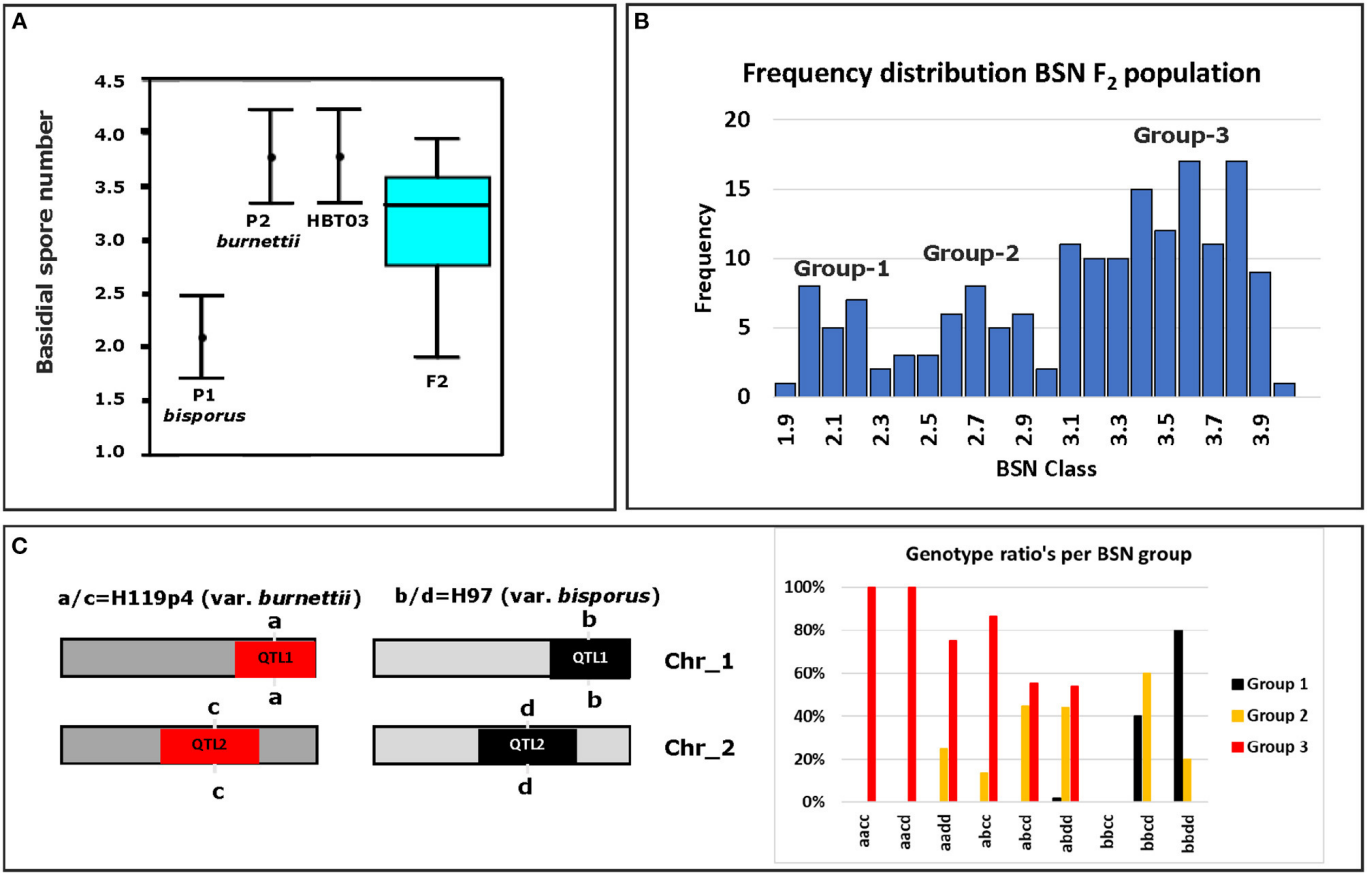
Basidial spore number (BSN) of parents, F_1_ and F_2_ intercross. **(A)** Averages and standard deviations of BSN of the parental lines (P1 [var. *bisporus* Horst U1] and P2 [var. *burnettii* 119/9]) and a box plot of the variation of BSN in the F_2_ intercrossed homokaryotic offspring of HBT03. The bar in the box indicates the median BSN. **(B)** Frequency distribution of average spore number of the F_2_ intercrossed population. Three groups can be discriminated with variation of BSN around 2.1, 2.8, and 3.6. Bin size is 0.1. **(C)** Left, the schematic representation of the genotypes on chromosomes 1 and 2 for the SNP-marker at the peak of each QTL for both parental homokaryons. Right, the frequency distribution of genotypes of the F_2_ intercrossed population normalized for the number in each class.

**Figure 4:**
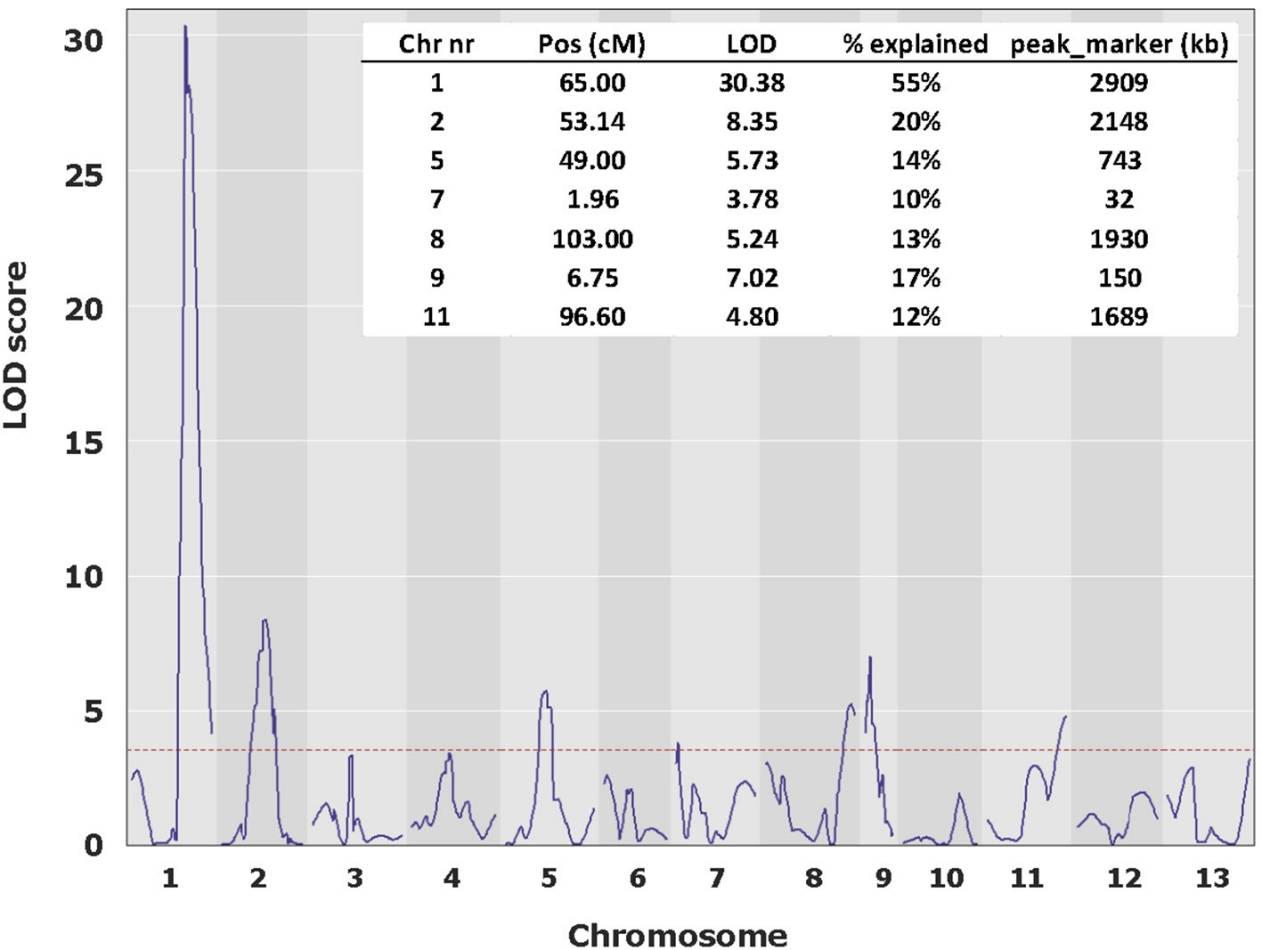
QTL plot for BSN along the 13 chromosomes. The major QTL, *Bsn*-QTL-1 on chromosome 1 peaks at a LOD value of 30 and Bsn-QTL-2 on chromosome 2 peaks at a LOD value of 8. LOD threshold (*p* < 0.05) is 3.52.

To map the average basidial spore numer (BSN), 42 individuals of the mapping population were intercrossed generating 230 F_2_ hybrids. Since homokaryons can only be crossed with homokaryons carrying compatible mating types to generate mushroom forming heterokaryons, no segregation will be seen for loci around the Mat A locus on chromosome 1. Previous research has shown that a major locus for the basidial spore number (*Bsn*) is located on the right site of chromosome 1, ~2 Mb away of the Mat A locus that is positioned on the left site of chromosome 1 (Foulongne-Oriol et al., 2010). The absense of visible segregation in the Mat A region is thus not hiding this major QTL for *Bsn*. Fruiting bodies of the parental lines, the intervarietal HBT03 hybrid and 173 intercrosses (F_2_) were assessed for the number of spores per basidium as described in M&M. Some 25% of the intercrosses did not fruit and although fruiting bodies of all other F_2_ hybrids did form spore bearing basidia, the spores were not shed. This is likely due to inbreeding depression, often seen in *A. bisporus* (Xu, 1995; Sonnenberg et al., 2017). The basidial spore numbers could be assessed for nearly all hybrids that produced fruiting bodies. The parental lines (Horst U1 and 119/9) showed a clear opposite phenotype with an average basidial spore number of 2.10 and 3.62, for var. *bisporus* and var. *burnettii*, respectively (Figure 3A; Table 1A). The HBT03 intervarietal hybrid has an average spore number of 3.78 showing that the trait *Bsn* is dominant. HBT03 has a higher percentage 4-spored and lower percentage 3-spored basidia than the parental var. *burnettii* line. By assuming that 2-, 3- and 4-spored basidia produce zero, two and four homokaryotic SSI per basidium (Kerrigan et al., 1994), the number of expected homokaryotic SSI among all spores is 10%, 90% and 94% for var. *bisporus*, var. *burnettii* and the HBT03 intervarietal hybrid, respectively. The F_2_ intercross showed a range of average basidial spore numbers varying from 1.88 up to 3.95 (Figure 3A). A histogram plot for the average basidial spore number of the F_2_ suggests three groups with averages around 2.1, 2.8 and 3.6 (Figure 3B; Table 1B) with predominantly 2, 3 and 4-spored basidia, respectively. Interval mapping (IM) revealed seven QTL's with the two largest QTL on chromosomes 1 (*Bsn-*QTL-1) and chromosome 2 (*Bsn-*QTL-2) with a maximal LOD value of 30 and eight, respectively (Figure 4). *Bsn-*QTL-1 explained 55% and *Bsn-*QTL-2 explained 20% of the variation in BSN. The frequency of the genotype at the peak of the two major QTL in each of the three groups, normalized for the number of individuals in each class, supports this grouping (Figure 3C). Group 3 is represented by either homozygous var. *burnettii* genotypes for QTL-1 and homo/heterozygous var. *burnettii* genotypes for QTL-2. Heterozygosity for QTL-1 combined with homo-heterozygous var. *bisporus* QTL-2 causes a shift toward Group 2. Group 1 is homozygous var. *bisporus* for QTL-1 and homo/heterozygous var. *bisporus* for QTL-2. It shows that both QTL contribute to the basidial spore number with a larger effect of QTL1.

**Table 1:** Phenotypic data of basidial spore number (BSN) for parents, the intervarietal hybrid **(A)** and the intercross **(B)**.

**(A)**			
	**Parents**		
	**Var**. ***bisporus***	**Var**. ***burnettii***	**Hybrid F1**
# spores/basidium	Horst U1	119/9	HBT03
2	92.00%	3.67%	
3	4.65%	30.33%	
4	2.66%	66.00%	
Average BSN	2.10 ± 0.38	3.62 ± 0.56	3.78 ± 0.44
% Homokaryons	9.50%	89.60%	94.30%
**(B)**			
**# spores/basidium**	**Group-1**	**Group-2**	**Group-3**
1	3.42%	0.86%	0.15%
2	80.36%	30.30%	5.19%
3	15.00%	54.92%	36.65%
4	0.96%	13.92%	57.99%
5	0.01%	0.00%	0.02%
Average BSN	2.13	2.81	3.52
Range	1.9–2.5	2.5–3.1	3.1–4.0

***(A)** Phenotypic data of basidial spore number (BSN) for the parents, the intervarietal hybrid and the intercross. The parental varieties show a clear opposite average basidial spore number and the intervarietal hybrid shows that the elevated spore number of the var. burnettii is dominant. **(B)** The distribution of spore numbers per basidia in the three groups, with each group a distinct mode with predominantly 2, 3, and 4-spored basidia, respectively*.

### CO Landscape of Parental Lines and the HBT03 Intervarietal Hybrid

**Figure 5:**
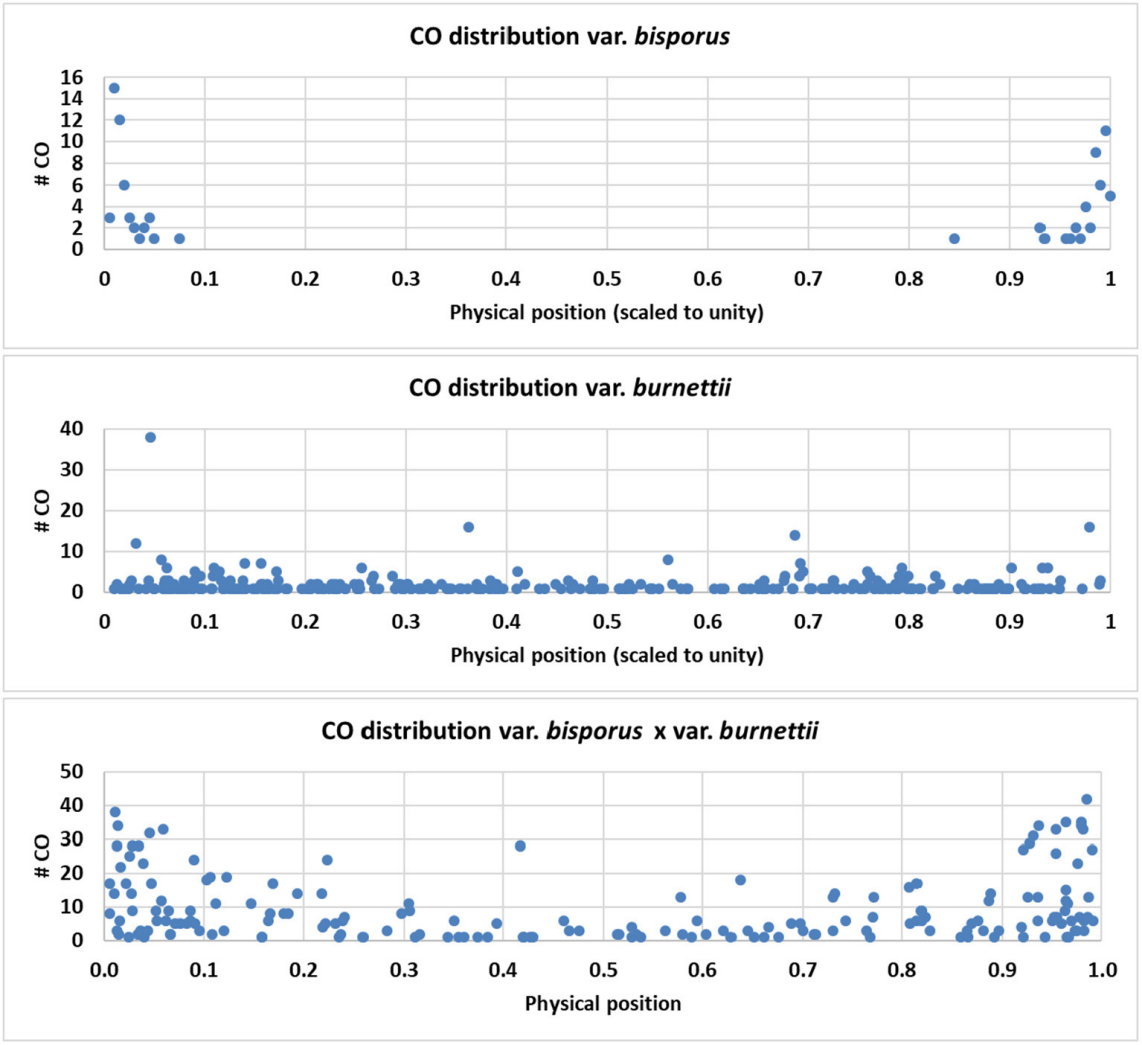
CO distribution of the parental lines Horst U1 (var. *bisporus*), 119/9 *(*var. *burnettii*), and the intervarietal hybrid HBT03. CO positions were calculated as described in MandM, scaled according to a standard chromosome length of one, and subsequently plotted against the physical position on the standard chromosome. A clear contrasting phenotype is seen in the parental lines and an intermediate pattern in the intervarietal hybrid.

In order to compare the CO position along chromosomes for the parental lines (Horst U1 and 119/9) and the HBT03 intervarietal hybrid, data on CO positions available from this and previous studies were used as described in M&M. CO positions for all data sets were scaled according to a standard chromosome length of one, and subsequently plotted against the physical position on the standard chromosome. The plots show a clearly different CO distribution of the parental lines, i.e., a restriction of CO to chromosome ends for var. *bisporus* and an even distribution for var. *burnettii*. The intervarietal hybrid HBT03 shows an intermediate pattern with an elevated number of CO toward chromosome ends (Figure 5). The data from the parental line var. *burnettii* were derived from Genotyping by Sequencing data which generated many markers at a high density. However, data could only be used for chromosomes 1, 3, 10, and 11 because other chromosomes were highly homozygous (Sonnenberg et al., 2020). Some regions of chromosomes 1, 10, and 11 also contained homozygous regions, generating gaps without markers of which some caused a high amount of CO between bordering markers, as expected. The intermediate CO phenotype of the intervarietal hybrid might indicate that the positioning of CO is a complex, polygenic trait.

### Generating Offspring of the Mapping Population

In order to assess the influence of the genotype of individuals of the HBT03 offspring mapping population on the CO position, offspring has to be generated from each of the individuals of the mapping population. Thus, each of the individuals of the mapping population must be crossed with a compatible homokaryon to generate mushrooms (F_2_) from which in turn the haploid offspring can be genotyped to assess CO distribution. Two types of hybrids can be made to generate (haploid) offspring, intercrosses and outcrosses. Intercrossing compatible homokaryons of the mapping population would generate the highest variation in phenotypes since the hybrids would display a variation of homo- and or heterozygosity of genes involved. Disadvantages of intercrosses are the limited compatible crossings that can be made as only 50% of the individuals are compatible, no segregation can be observed at (or close nearby) the mating type locus, and many genome areas will be homozygous and hide COs. An intercross/selfing will thus not be useful. Advantages of outcrossing are a higher polymorphism between the parental genotypes allowing a better estimate of CO positions. In addition, there are no limitations in mating type compatibility when a suitable tester homokaryon is chosen, i.e., a different mating type than the mating types present in the mapping population. However, outcrossing of the mapping population with a var. *bisporus* or var. *burnettii* will shift the CO distribution to either the chromosome ends or toward a more even distribution over the chromosomes, respectively, reducing the variation in the phenotype. Two types of hybrids were generated from the mapping population, a cross with H39 (the other nucleus of the *bisporus* variety Horst U1 [H39 × H97]), or with H119p1 (the other nucleus of the *burnettii* variety 119/9 [H119p1 × H119p4]; see Figure 1). Fruiting of the different types of hybrids showed that outcrossing with H119p1 resulted in severe inbreeding depression. Most heterokaryons did not fruit, and those that produced mushrooms generated spores with a very low germination rate. The observed inbreeding depression might be due to the high similarity between the genomes in the tester homokaryon *burnettii* strain H119p1 and the H119p4 parent of the mapping population (Sonnenberg et al., 2020). This outcross was therefore not useful. Outcrosses with H39 displayed normal fruiting bodies that shed spores, and of which spores had a normal gemination rate. Therefore, H39 var. *bisporus* provided the best option to be used as a tester homokaryon to generate haploid offspring from individuals of the HBT03 mapping population, in which COs could be assessed.

### Marker Design

Since the phenotype (CO landscape) had to be assessed in offspring of the individuals of the mapping population x var. *bisporus* H39, a shift was expected from the CO landscape phenotype of the HBT03 intervarietal hybrid to that of var. *bisporus*. To estimate CO positions and to keep costs affordable, one marker was chosen as far as possible toward each chromosome end and a second marker 150-200 kb inwards from the chromosome end (Supplementary Figure 2). The latter function as the “border” between End-CO and Middle-CO. CO within marker pairs left and marker pairs right on each chromosome will thus be registered as CO at ends and CO between the two “border” markers as CO in the middle. The fraction of individuals with a CO between the different markers were calculated as described in M&M. In order to have a correct estimation of COs per region of each chromosome, markers should be located as much as possible on similar positions for each chromosome. Chromosomes 3, 8, and 9 were excluded as no suitable SNP markers could be found either at extreme ends or in the area 150–200 kb away from the chromosome ends. For all the remaining chromosomes, markers were generated in a similar position as much as possible, although especially at chromosome ends equal positioning was suboptimal (Sonnenberg et al., 2016; Supplementary Table 2).

### Phenotyping

Phenotyping of the CO landscape in offspring of the mapping population is a very laborious task since from each crossed individual of the mapping population, enough haploid offspring must be isolated and genotyped to assess the effect of the genotype on the CO landscape. The effort will depend also on how many homokaryons will be present amongst the F_2_ offspring. We estimated that an F_2_ offspring of 50 homokaryons would suffice for phenotyping of a single F_1_ individual. The number of homokaryons that is expected in the F_2_ offspring, and thus the amount of SSI that has to be screened for the presence of one or two nuclear types, depends on the genotype of the *Bsn* locus in the F_2_ hybrids. Selection of individuals in the mapping population with a *Bsn-*QTL1 genotype of var. *burnettii* would increase the number of homokaryons among offspring considerably (see paragraph “QTL mapping of BSN”) and thus reduce labor costs. This requires, however, that the CO landscape phenotype is not linked to the *Bsn*-QTL1 locus. To test if the major QTL (*Bsn-*QTL1) is linked to CO landscape, five SSIs of the HBT03 mapping population were selected having the var. *bisporus* allele and 5 with the var. *burnettii* allele. A cross of these individuals with H39 will generate F_2_ heterokaryons of which five are homozygous (var. *bisporus*) for *Bsn*-QTL1 and five heterozygous for *Bsn-*QTL1 (intervarietal). A range of 28–47 homokaryotic SSIs of each F_2_ heterokaryon were genotyped as described. Only three chromosomes (chromosomes 1, 4, and 13) were genotyped to obtain an impression of the CO positions. To see if the *Bsn*-QTL1 locus is linked to CO position, the fraction of CO in the middle of the chromosome (#CO between markers 150–200 kb away from chromosome ends divided by CO between all markers) were calculated for the three chromosomes. Although there is a difference in the fraction of CO in the middle between the hybrids homo- or heterozygous for *Bsn*-QTL1, a *t*-test showed that this difference was not significant (Supplementary Table 3).

### QTL Mapping of CO Position

**Figure 6:**
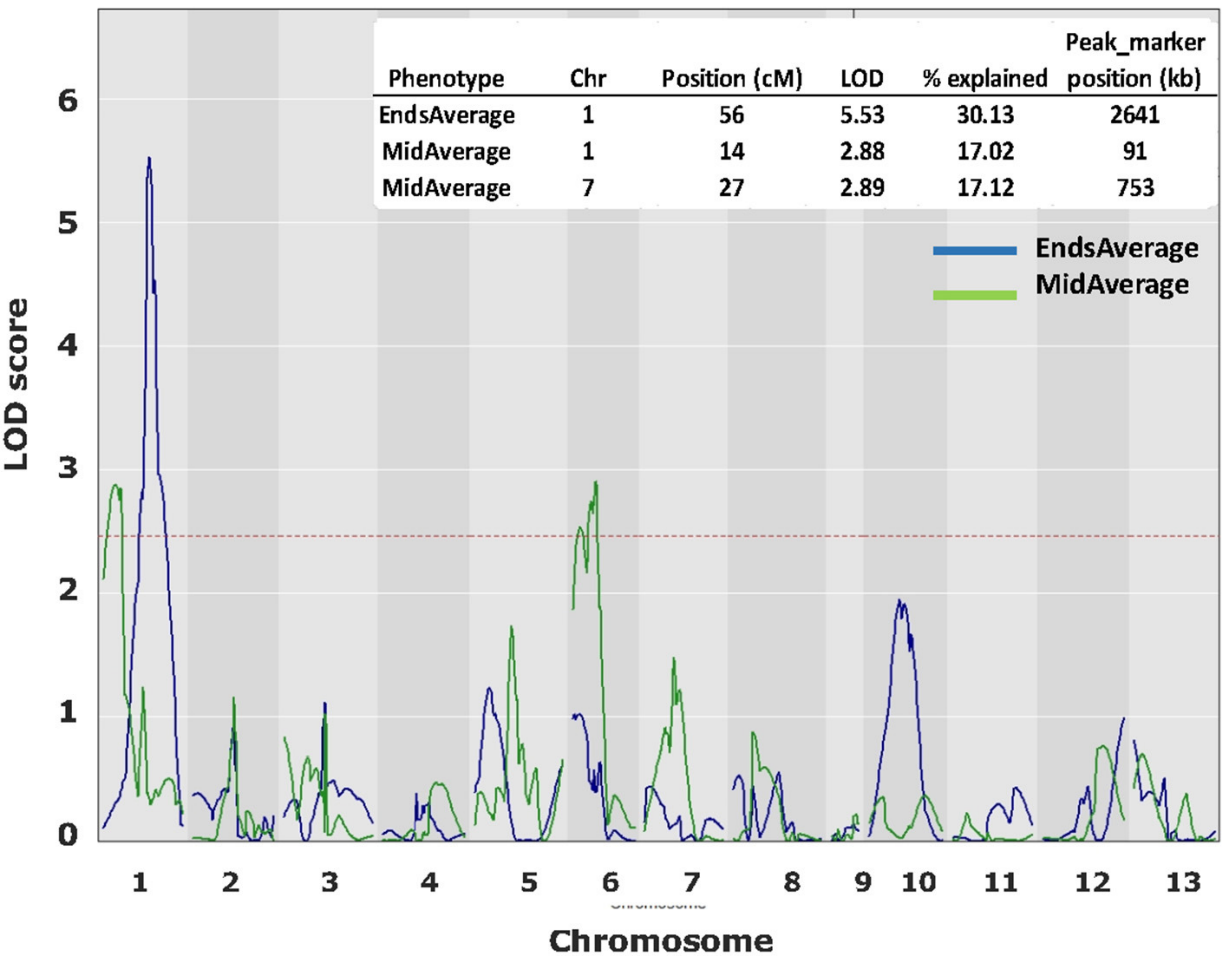
QTL plots for CO landscape over 13 chromosomes. In blue the QTL plots for average CO at chromosome ends and in green the QTL plots for CO at the middle of chromosomes. QTL analysis was done with R (package QTL2) using Haley–Knott Regression to identify significant QTLs. LOD threshold (< 0.1): 2.49.

From the HBT03 mapping population, 54 homokaryons with the var. *burnettii Bsn*-QTL1 allele and 17 with the var. *bisporus Bsn*-QTL1 allele were selected, a just manageable 71 in total. These, crossed with H39, generated a majority of hybrids heterozygous for *Bsn*-QTL1 and thus increased the proportion of homokaryotic spores in F_2_ offspring considerably. The inclusion of 17 *Bsn-*QTL1 var. *bisporus* homokaryons (generating 17 heterokaryons homozygous for var. *bisporus Bsn*-QTL1) should reveal if there is a strong linkage between this QTL and CO position. In total more than 20,000 F_2_ single spore offspring had to be isolated and genotyped for their mating type (distinguishing heterokaryons from homokaryons) to reach the required 50 homokaryotic offspring for each F_1_ individual to assessing the CO landscape. COs within the marker pairs at each end of the chromosome and CO between the two “border” markers represent COs at chromosome ends and COs in the middle of chromosomes, respectively. The latter pair will not detect double CO in the middle of the chromosome but since most of the CO have shifted toward the chromosome ends in the F_2_ offspring due to outcross with H39, these are relatively rare. The calculation of the frequency of COs at the end and in the middle of each chromosome was done as described in M&M. As expected, the frequencies of the End-fractions and Middle-fractions clearly showed that outcrossing with H39 shifts the phenotype toward the var. *bisporus* (Supplementary Figure 3), resulting in a limited variation of the phenotype. QTL interval mapping revealed a QTL on chromosome 1 for EndsAverage and 2 QTLs for MidAverage, one on chromosome 1 and one on chromosome 6 (Figure 6). These QTLs were just above the threshold level and covered a broad area of the chromosome. The average of the MidAverage of the significant QTL on chromosome 1 and 6 were higher for the var. *burnettii* genotype than for the var. *bisporus* genotypes in these areas, as expected. The average of the EndsAverage for QTL on chromosome 1 had also a higher value for the genotypes of var. *burnettii* compared to the var. *bisporus* genotypes.

## Discussion

Unraveling the genetic basis for the extreme differing CO landscapes of two varieties of *A. bisporus* will have an enormous impact on breeding efficiency of this commercially important edible mushroom. If the mechanism can be unraveled or if homologs can be found in plant genomes with similar functions, this knowledge might also have relevance for plant breeding. A number of genes involved in CO position and frequencies have been studied in plants (Blary and Jenczewski, 2019; Fayos et al., 2019; Taagen et al., 2020). Interference of expression of these genes does not change CO positions in an extreme way as seen in the button mushroom and it is thus unlikely that homologs of these genes are involved in the different CO landscapes found in *A. bisporus*. In *Coprinopsis cinerea*, expression studies have revealed a large number of genes up or down regulated during meiosis (Burns et al., 2010; Anderson et al., 2012). Meiosis occurs synchronously in basidial cells in this basidiomycete, allowing tissue collection at different meiotic stages and comparison of gene expression. Meiosis does not occur synchronously in *A. bisporus* and such an approach would not be possible in this organism. The best option is thus segregation analysis in crosses between the two *A. bisporus* varieties. Segregation analysis for the complex trait CO landscape has not been done before and, not surprisingly, we have encountered a number of hurdles. For a trait that can only be assessed in offspring of a mapping population and that needs a precise positioning of markers in a large number of samples, we were restricted in the type and amount of data that could be generated. Due to the inability to especially position markers at the extreme ends of chromosomes only data from CO between the middle markers were reliable and even for these markers no data for all chromosomes could be generated. In addition, double crossovers might have been missed and reduced the robustness of the data. In order to reduce the labor costs for mapping CO positions, the majority (72%) of the selected individuals of the mapping population had the BSN genotype of var. *burnettii* for the major *Bsn*-QTL thus enhancing the number of homokaryons. Although comparison of a small sample indicated that this region is not likely linked to CO landscape, we cannot exclude that this biased selection has had an influence. A QTL was found for CO at chromosome ends (EndsAverage) in the *Bsn*-QTL1 region with the explaining genotypes from var. *burnettii*. That is not what is expected since the var. *bisporus* has a higher average for CO at chromosome ends. This indicates that the data for CO at ends might not be reliable, probably due to the fact that markers could not always be generated at extreme ends of chromosomes and thus CO might have been missed. An alternative explanation could be an unknown epistatic effect between relevant alleles from var. *bisporus* and var. *burnettii* which causes this unexpected result. For the two QTLs found for CO at the middle the expected genotype is var. *burnettii*. The genomic variation in the QTL regions identified for CO landscape explain just a small part of the phenotypic variation and the QTL cover broad areas on chromosomes. Alignment of the annotated regions for QTL of both CO landscape and BSN did not reveal clear differences in presence or absence of genes, indicating that the genetic differences are more subtle, likely allelic.

### A New Approach

**Figure 7:**
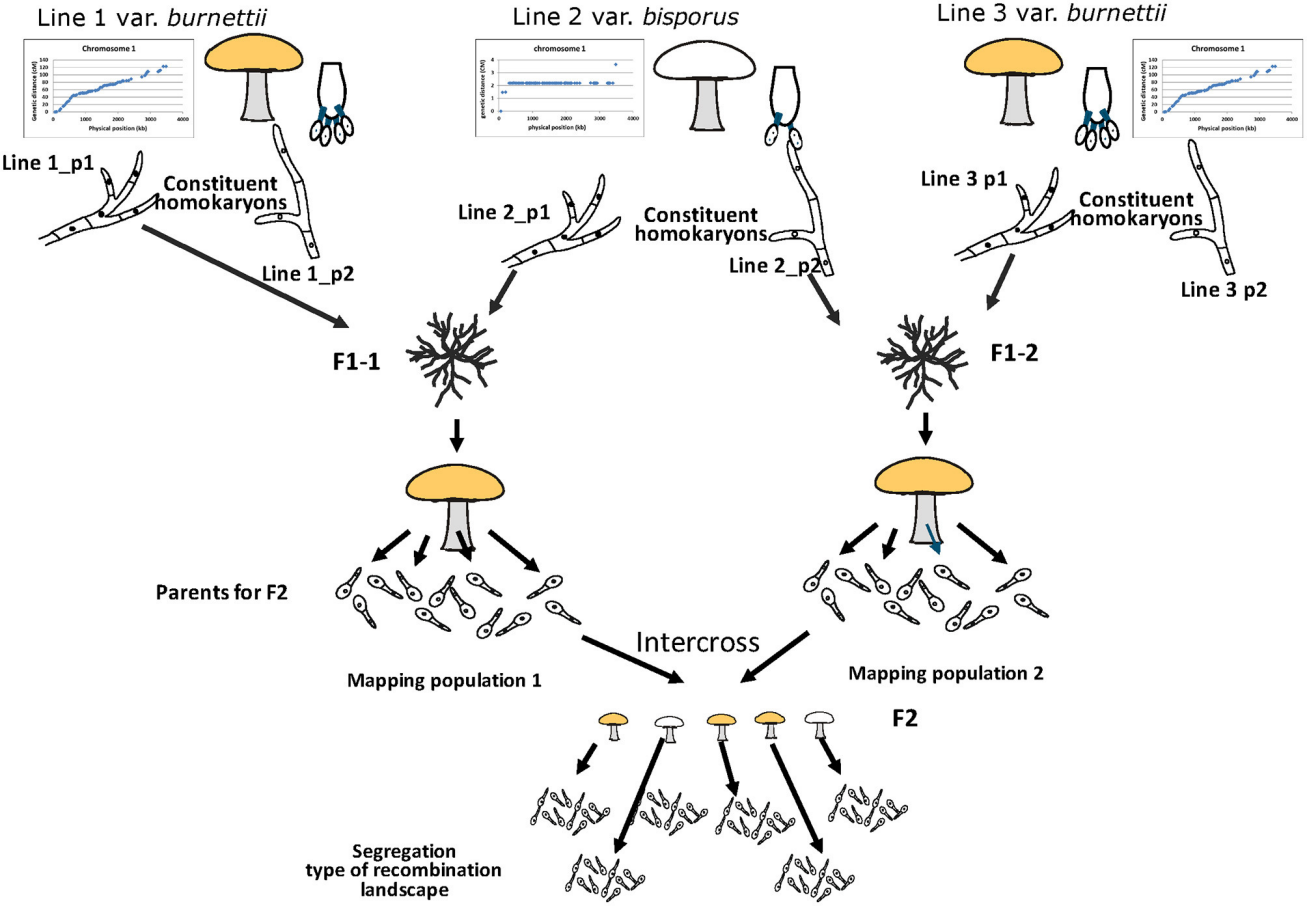
Proposed new approach for mapping BSN and CO landscape in *A. bisporus*. Two genetically unrelated mapping populations are generated, both segregating for BSN and CO landscape. In this way crosses can be made between these two populations simulating an intercross within one population without the problems of homozygosity (lack of segregating markers and inbreeding depression) and with the advantage of a higher allele frequency that will strengthen the QTLs. The mushrooms of the intercross (F_2_) can be used to assess BSN and homokaryotic offspring of this F_2_ cross can be resequenced to assess precisely the CO positions without the need to isolate first the homokaryons within the single spore isolates.

One of the main hurdles has been the limited possibility for selecting a suitable tester homokaryon to generate haploid offspring of the mapping population. The only option (outcross with a var. *bisporus* homokaryon) has resulted in a very narrow range of phenotypes. All CO clustered around chromosome ends with just a small shift toward the middle of chromosomes. The best option to obtain a high variation in phenotypes, i.e., intercross/selfing, was impossible due to lack of genetic polymorphism that hampers positioning of CO, a lack of segregation in the mating type region, and inbreeding depression. We have tested in the meantime another var. *burnettii* strain and have seen that, next to being mainly tetra-sporic, this strain also displays a more or less even distribution of CO over chromosomes. This offers a possibility to generate two genetically unrelated mapping populations, both offspring of a cross between a var. *bisporus* and a var. *burnettii* homokaryon (Figure 7) and thus segregating both for the same phenotypes. All individuals of one mapping population can be crossed with all individuals of the other mapping population due to differences in mating type. The mushrooms of this F_2_ population can be used directly to assess the average spore number and map BSN. The homokaryotic offspring of each F_2_ cross can be used to assess CO positions. This simulates a selfing for a population heterozygous for BSN and CO landscape. A prerequisite is that genes involved in CO positioning are identical within each of the varieties which seems likely since other var. *bisporus* (Sonnenberg et al., 2016) and other var. *burnettii* strains [including the one used by Foulongne-Oriol and colleagues (Foulongne-Oriol et al., 2010)] have the same respective typical CO landscape. Another hurdle has been the generation of enough reliable markers on desired positions to detect CO. Especially markers at chromosome ends have been a problem. Previous research has shown that resequencing technologies generate enough power to detect CO at extreme ends of chromosomes (Sonnenberg et al., 2016, 2020). If two different mapping population are crossed to study segregation of CO positions, as mentioned above, a shift in phase for marker pairs relative to the parental haplotypes must be detected. This requires knowledge of the genotypes for all markers in the parents which consists of a mosaic of the four original parental homokaryons (Figure 7). The haplotypes of the F_2_ parents can in principle be reconstructed from bulked resequenced data of its homokaryotic offspring (Peñalba and Wolf, 2020). After all, the vast majority of adjacent marker pairs in the homokaryons within a set of offspring will be the same as one of the two parental haplotypes and reconstruction will be facilitated by the high genomic collinearity of different homokaryons. This requires that the length of the pair-end reads exceed the gaps between adjacent SNP markers. Previously obtained data have shown a high SNP density between H119p4 and a constituent nucleus of another var. *burnettii* strain (>88% of adjacent SNP have distance less than 300 bp; Supplementary Figure 4). The vast majority of the pair-end reads of homokaryotic offspring of a cross between such homokaryons will thus contain at least two adjacent SNP markers. Pair-end resequencing, bulked per homokaryotic set of offspring, will thus generate data to reconstruct the parental phase and allows the positioning of CO. Alternatively, individuals of the two mapping populations can be resequenced to reconstruct the genomes in the mapping population. The advantage is that these data allow a dedicated selection of individuals to be crossed assuring that each has at least one CO at every chromosome and that all four parental genomes are equally represented. The construction of linkage maps, and thus assessment of recombination, and reconstruction of haplotypes can even be done with low-coverage resequencing data (Rastas, 2017; Rubinacci et al., 2021). The resolution of the sequencing data needed to detect COs will depend on the genome coverage (100–1,000 X) and the number of homokaryons pooled. Another advantage of sequencing bulked samples is that there is no need to select for homokaryons within sets of spore prints. For CO assessment in bulked samples it doesn't matter if gametes are derived from the same or different meiotic events. Both the intercross of unrelated mapping populations and precise assessment of CO positions will certainly narrow the QTL regions not only for CO landscape but also for BSN. The main genetic locus for BSN has been previously mapped to chromosome 1 (Imbernon et al., 1996; Foulongne-Oriol et al., 2010). Segregation of the BSN phenotype was studied using haploid offspring of a var. *bisporus* × var. *burnettii* hybrid outcrossed with var. *bisporus* homokaryons, as was done here for mapping the CO landscape. Imbernon et al. observed a bimodal segregation with peaks of ASN at 2.5 and 3.5. Because of the continuous distribution of ASN and the bimodal character they concluded that *Bsn* locus on chromosome 1 was the main determinant for spore number trait. Our intercross allowed the segregation of all possible alleles involved. A frequency plot of the average basidial spore number (ASN) clearly shows three groups, also indicating the power of an intercross compared to outcrossing. As in the Foulongne-Oriol et al. (2010), in our map the main peak of the BSN locus is located on chromosome 1 and is separated from the mating type locus by more than 2 Mb. In the intercross population and the outcross with the var. *burnettii* homokaryon (H119p1) we observed different inbreeding depressions. While in both types of populations some hybrids did not fruit at all, in the first population we saw spore formation, but these spores were not shed, whereas in the latter population spores were shed but did not germinate. Both type of hybrids differs in regions that are homozygous, and this might indicate the presence of deleterious genes in these regions. Identifying the responsible genes will be relevant to reduce inbreeding depression. By retaining these regions heterozygous in repeated backcrosses, it will reduce inbreeding depressions and thus facilitating introgression breeding.

Mushroom breeding is in its infancy if compared to plant breeding. Most research published on breeding is on detecting QTL in offspring of biparental crosses (crosses between homokaryotic single spores or crosses between recovered homokaryons/haplotypes of different strains). The segregation of phenotypes is assessed by intercrossing the mapping population or outcrossing with a tester line. As shown in this paper, these strategies can generate serious problems in the precision of QTL mapping and thus the generation of superior varieties. Not surprisingly, hardly any publications can be found on a successful introduction of QTL mapped traits into commercial cultivars. The above described crossing of two genetically unrelated populations segregating for the same traits is uncommon in mushroom breeding and has potential to improve the present breeding strategies. These types of crosses enhance the allele frequency and phenotypic variation thus increasing the power and precision of QTL location (Cavanagh et al., 2008; Galeano et al., 2011). In addition, verification of QTL in different backgrounds will also elucidate the consistency of a QTL and, if consistent, enables its introduction in different genetic backgrounds and consequently has a broader application in breeding. Last but not least, this strategy can also reduce the problem of inbreeding. After generation of markers linked to QTL's, relevant individuals of each population can be selected and backcrossed with parents of the genetically unrelated variety. Introgression to some extend can thus be done in recipient varieties without too much inbreeding depression since the first cross will avoid inbreeding. As a last step, the introgressed varieties can be crossed to combine the best alleles and the resulting varieties screened for performance.

## Data Availability Statement

The raw data on BSN, the genotypes of the mapping population and selection for phenotyping the recombination landscape and the input for QTL mapping of the recombination landscape are available at: https://doi.org/10.4121/14695425.

## Author Contributions

NS-T, BL, and PH performed the experiments. BL, NS-T, and AS performed data analysis. AS and AP wrote the manuscript. KS, JB, and RV performed reviewing and editing. AS supervised the project. All authors contributed to the article and approved the submitted version.

## Conflict of Interest

NS-T is employed by Ceradis B.V. after the finish of this research. The remaining authors declare that the research was conducted in the absence of any commercial or financial relationships that could be construed as a potential conflict of interest.

## Publisher's Note

All claims expressed in this article are solely those of the authors and do not necessarily represent those of their affiliated organizations, or those of the publisher, the editors and the reviewers. Any product that may be evaluated in this article, or claim that may be made by its manufacturer, is not guaranteed or endorsed by the publisher.

## References

[B1] Anderson E. Burns C. Zolan M. E. (2012). Global gene expression in coprinopsis cinerea meiotic mutants reflects checkpoint arrest. G3 (Bethesda) 2, 1213–21. 10.1534/g3.112.003046 23050232 PMC3464114

[B2] Blary A. Jenczewski E. (2019). Manipulation of crossover frequency and distribution for plant breeding. Theor. Appl. Genet. 132, 575–592. 10.1007/s00122-018-3240-1 30483818 PMC6439139

[B3] Broman K. W. Gatti D. M. Simecek P. Furlotte N. A. Prins P. Sen S.. (2018). R/qtl2: software for mapping quantitative trait loci with high-dimensinal data multi-parent popukations. Genetics 211, 495–502. 10.1534/genetics.118.301595 30591514 PMC6366910

[B4] Burns C. Stajich J. E. Rechtsteiner A. Casselton L. Hanlon S. E. Wilke S. K.. (2010). Analysis of the basidiomycete coprinopsis cinerea reveals conservation of the core meiotic expression program over half a billion years of evolution. PLoS Genet. 6:e1001135. 10.1371/journal.pgen.1001135 20885784 PMC2944786

[B5] Callac P. Billette C. Imbernon M. Kerrigan R. W. (1993). Morphological, genetic, and interfertility analyses reveal a novel, tetrasporic variety of *Agaricus bisporus* from the Sonoran desert of California. Mycologia 85, 835–851. 10.1080/00275514.1993.12026340

[B6] Callac P. Jacobé de Haut I. Imbernon M. Guinberteau J. Desmerger C. Theochari I. (2003). A novel homothallic variety of *Agaricus bisporus* comprises rare tetrasporic isolates from Europe. Mycologia 95, 222–231. 10.1080/15572536.2004.11833107 21156608

[B7] Cavanagh C. Morell M. Mackay I. Powell W. (2008). From mutations to MAGIC: resources for gene discovery, validation and delivery in crop plants. Curr. Opin. Plant Biol. 11, 215–221. 10.1016/j.pbi.2008.01.002 18295532

[B8] Elliott T. J. (1972). Sex and the single spore. Mushroom Sci. 8, 11–18.

[B9] Fayos I. Mieulet D. Petit J. Meunier A. C. Périn C. Nicolas A.. (2019). Engineering meiotic recombination pathways in rice. Plant Biotechnol. J. 17, 2062–2077. 10.1111/pbi.13189 31199561 PMC6790369

[B10] Foulongne-Oriol M. Spataro C. Cathalot V. Monllor S. Savoie J. M. (2010). An expanded genetic linkage map of an intervarietal *Agaricus bisporus* var. *bisporus*× *A. bisporus* var. burnettii hybrid based on AFLP, SSR and CAPS markers sheds light on the recombination behaviour of the species. Fungal Genet. Biol. 47, 226–236. 10.1016/j.fgb.2009.12.003 20026415

[B11] Galeano C. H. Fernandez A. C. Franco-Herrera N. Cichy K. A. McClean P. E. Vanderleyden J.. (2011). Saturation of an intra-gene pool linkage map: towards a unified consensus linkage map for fine mapping and synteny analysis in common bean. PLoS One 6:e28135. 10.1371/journal.pone.0028135 22174773 PMC3234260

[B12] Gao W. Baars J. J. Dolstra O. Visser R. G. Sonnenberg A. S. (2013). Genetic variation and combining ability analysis of bruising sensitivity in *Agaricus bisporus*. PLoS One 8:e0076826. 10.1371/journal.pone.0076826 24116171 PMC3792865

[B13] Grishaeva T. M. Bogdanov Y. F. (2018). Conservation of meiosis-specific nuclear proteins in eukaryotes: a comparative approach. Nucleus 61, 175–182. 10.1007/s13237-018-0253-8

[B14] Imbernon M. Callac P. Gasqui P. Kerrigan R. W. Velcko A. J. (1996). BSN, the primary determinant of basidial spore number and reproductive mode in *Agaricus bisporus*, Maps to Chromosome *I*. Mycologia 88, 749–761. 10.1080/00275514.1996.12026713

[B15] Kerrigan R. W. Imbernon M. Callac P. Billette C. Olivier J.-M. (1994). The heterothallic life cycle of *Agaricus bisporus* var. burnettii and the Inheritance of Its Tetrasporic Trait. Exp. Mycol. 18, 193–210. 10.1006/emyc.1994.1020

[B16] Kerrigan R. W. Royer J. C. Baller L. M. Kohli Y. Horgen P. A. Anderson J. B. (1993). Meiotic behavior and linkage relationships in the secondarily homothallic fungus *Agaricus bisporus*. Genetics 133, 225–236. 10.1093/genetics/133.2.225 8094696 PMC1205313

[B17] Pelham J. (1967). Tecgnique for mushroom genetics. Mushroom Sci. 6, 49–64.

[B18] Peñalba J. V. Wolf J. B. W. (2020). From molecules to populations: appreciating and estimating recombination rate variation. Nat. Rev. Genet. 21, 476–492. 10.1038/s41576-020-0240-1 32472059

[B19] Raper C. A. Raper J. R. (1972). Genetic analysis of the life cycle of *Agaricus bisporus*. Mycologia 64, 1088–1117. 10.1080/00275514.1972.12019354

[B20] Rastas P. (2017). Lep-MAP3: robust linkage mapping even for low-coverage whole genome sequencing data. Bioinformatics 33, 3726–3732. 10.1093/bioinformatics/btx494 29036272

[B21] Royer J. C. Hintz W. E. Kerrigan R. W. Horgen P. A. (1992). Electrophoretic karyotype analysis of the button mushroom, *Agaricus bisporus*. Genome 35, 694–698. 10.1139/g92-105

[B22] Rubinacci S. Ribeiro D. M. Hofmeister R. J. Delaneau O. (2021). Efficient phasing and imputation of low-coverage sequencing data using large reference panels. Nat. Genet. 53, 120–126. 10.1038/s41588-020-00756-0 33414550

[B23] Sonnenberg A. S. de Groot P. W. Schaap P. J. Baars J. J. Visser J. Van Griensven L. J. (1996). Isolation of expressed sequence tags of *Agaricus bisporus* and their assignment to chromosomes. Appl. Environ. Microbiol. 62:4542. 10.1128/aem.62.12.4542-4547.1996 8953726 PMC168281

[B24] Sonnenberg A. S. Gao W. Lavrijssen B. Hendrickx P. Sedaghat-Tellgerd N. Foulongne-Oriol M.. (2016). A detailed analysis of the recombination landscape of the button mushroom *Agaricus bisporus* var. bisporus. Fungal Genet. Biol. 93, 35–45. 10.1016/j.fgb.2016.06.001 27288752

[B25] Sonnenberg A. S. M. Baars J. J. P. Gao W. Visser R. G. F. (2017). Developments in breeding of *Agaricus bisporus* var. *bisporus*: progress made and technical and legal hurdles to take. Appl. Microbiol. Biotechnol. 101, 1819–1829. 10.1007/s00253-017-8102-2 28130632 PMC5309338

[B26] Sonnenberg A. S. M. Sedaghat-Telgerd N. Lavrijssen B. Ohm R. A. Hendrickx P. M. Scholtmeijer K.. (2020). Telomere-to-telomere assembled and centromere annotated genomes of the two main subspecies of the button mushroom *Agaricus bisporus* reveal especially polymorphic chromosome ends. Sci. Rep. 10:14653. 10.1038/s41598-020-71043-5 32887908 PMC7473861

[B27] Taagen E. Bogdanove A. J. Sorrells M. E. (2020). Counting on crossovers: controlled recombination for plant breeding. Trends Plant Sci. 25, 455–465. 10.1016/j.tplants.2019.12.017 31959421

[B28] Taylor J. Butler D. (2017). R Package ASMap: efficient genetic linkage map construction and diagnosis. J. Stat. Softw. 79, 1–29. 10.18637/jss.v079.i0630220889

[B29] Weijn A. Tomassen M. M. M. Bastiaan-Net S. Wigham M. L. I. Boer E. P. J. Hendrix E. A. H. J.. (2012). A new method to apply and quantify bruising sensitivity of button mushrooms. LWT 47, 308–314. 10.1016/j.lwt.2012.01.024

[B30] Xu J. (1995). Analysis of inbreeding depression in *Agaricus bisporus*. Genetics 141, 137–145. 10.1093/genetics/141.1.137 8536962 PMC1206712

[B31] Young J. A. Hyppa R. W. Smith G. R. (2004). Conserved and nonconserved proteins for meiotic DNA breakage and repair in yeasts. Genetics 167, 593–605. 10.1534/genetics.103.023762 15238514 PMC1470912

