# Supplementary material for: Mapping Recombination Landscape and Basidial Spore Number in the Button Mushroom Agaricus bisporus
Source: Front Fungal Biol. 2021 Aug 20;2:711330. doi: 10.3389/ffunb.2021.711330 (PMC10512247; doi:10.3389/ffunb.2021.711330)

**Supplemental Data**

Figure S1. **Genotypes of the homokaryotic individuals of the mapping population selected for mapping CO landscape.** As in the total mapping population, this selection also shows a skewed segregation for chromosome 2 and especially chromosome 3, with an overrepresentation of the H97 genotype.


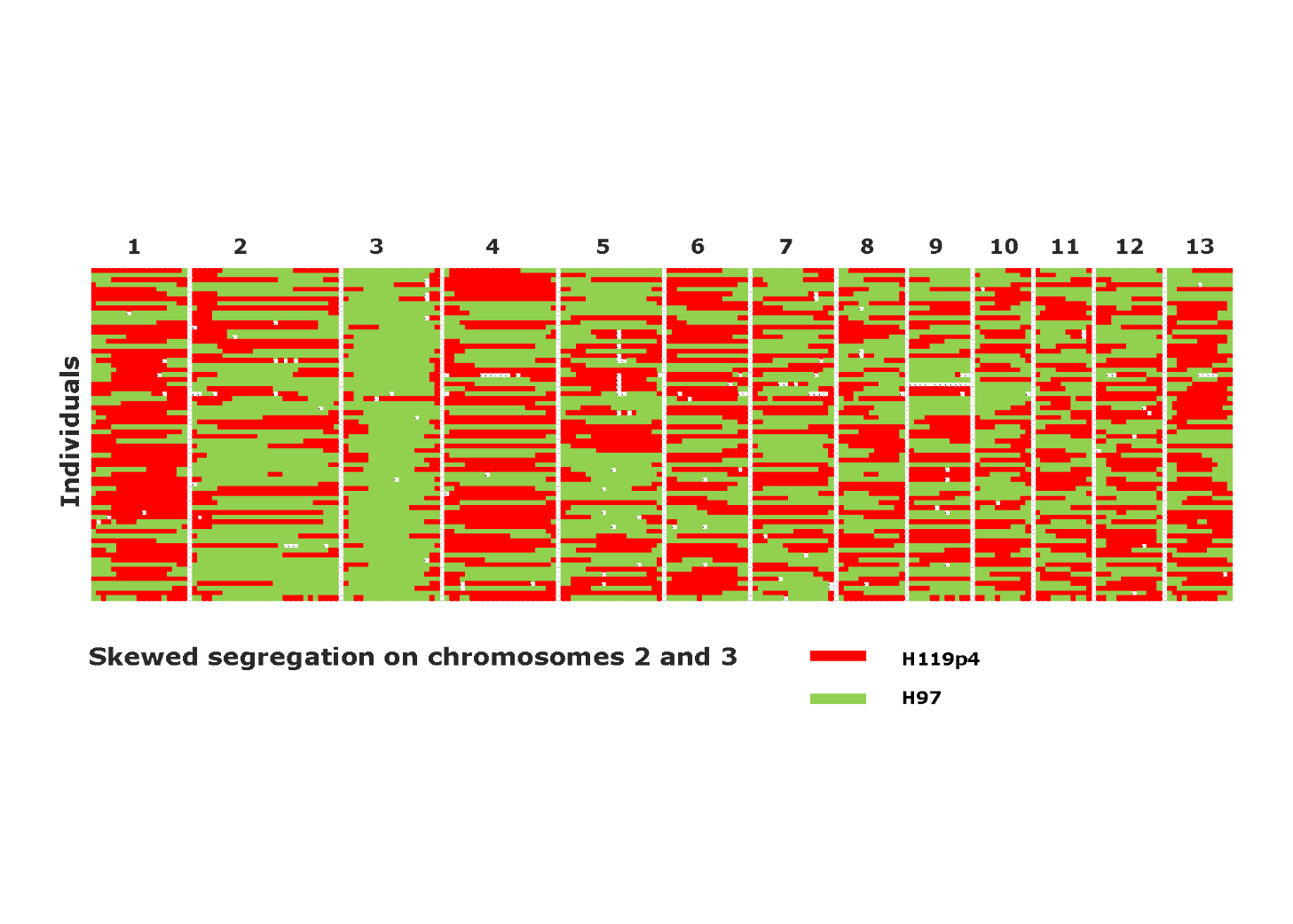


Figure S2. **Schematic representation of position of markers used to assess the CO position in offspring of the outcrossed mapping population**. CO at chromosome ends were scored by calculating the relative frequencies of crossovers between end markers (a SNP as far as possible at the chromosome end and a SNP 150-200 bp from the chromosome end, the latter also designated as a “border” marker). CO in the middle were scored by calculating the relative CO frequencies between the middle markers (between the 2 “border” marker on each chromosome).


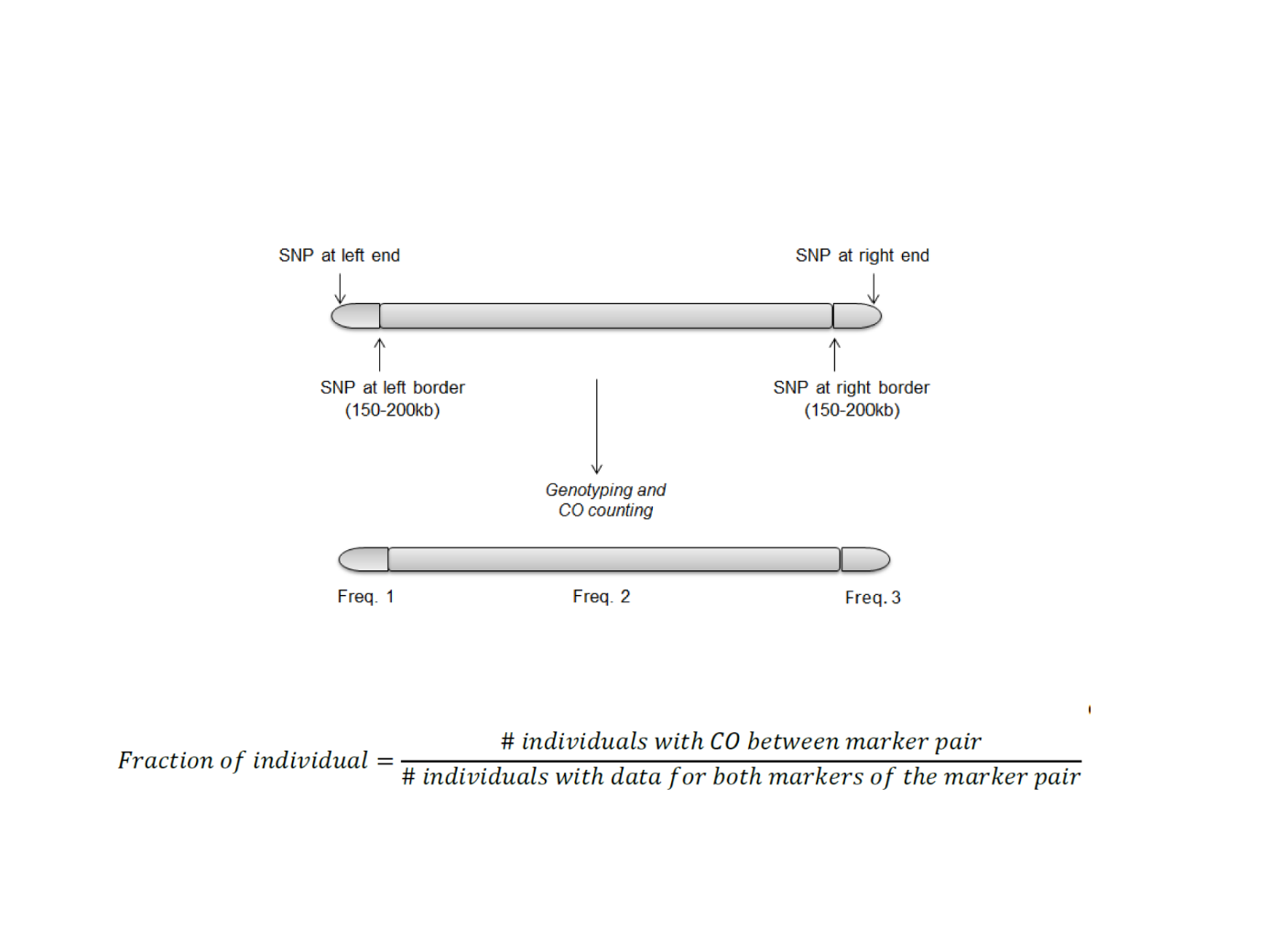


Figure S4. Frequency distribution of the average distance between adjacent SNPs for 2 different var. *burnettii* homokaryons (H119p4 and another var. *burnettii* homokaryon). Data are calculated only for chromosome 2 as an example.


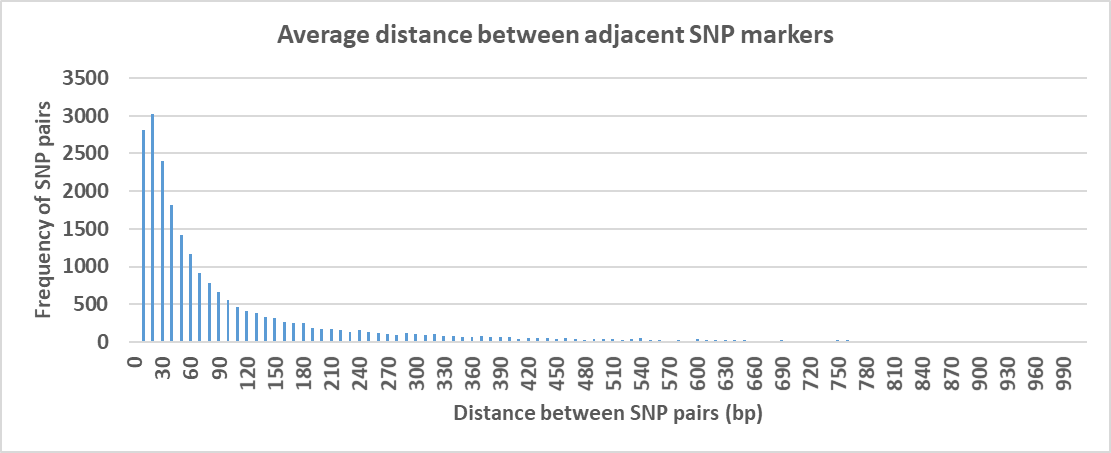


Figure S3. Distribution of CO frequencies at chromosome ends or in the middle of chromosomes after outcrossing of the mapping population with var. *bisporus homokaryon* H39. A clear shift is seen towards chromosome ends relative to the CO’s of the F_1_ (HBT03). The CO frequency data for Ends and Middle do not always add up to 1 (see Average Ends and Average Middle for F_1_). The reason for this is that only if for all data for chromosomes ends and middles are available for the same chromosomes, these data must add up to 1. That is, however, not the case due to missing data.


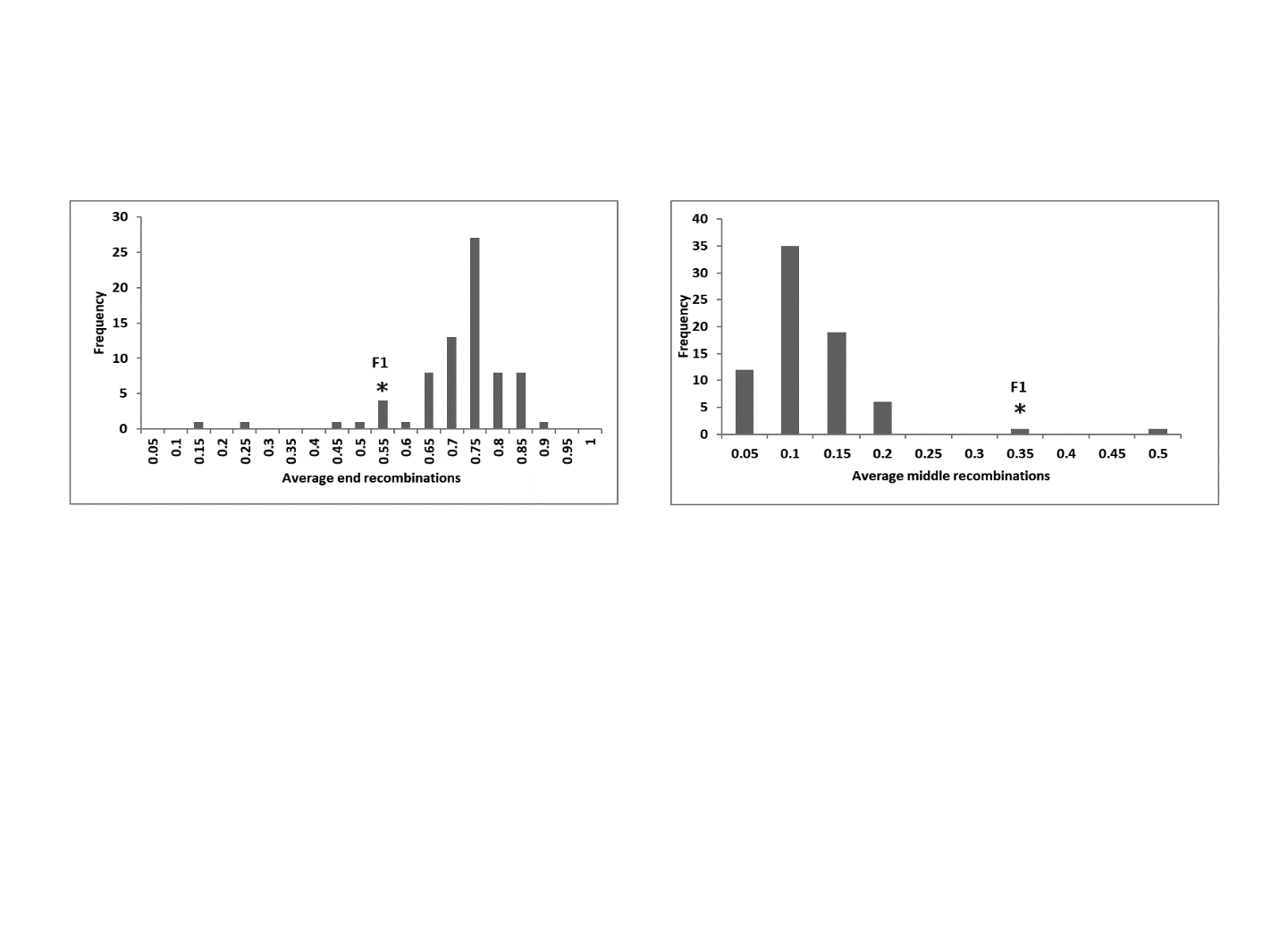


Table S1. Statistics of the linkage map of a segregating population derived from a cross between var. *bisporus* (homokaryon H97) and var. *burnettii* (var. *burnettii*).

Table S2. Positions of the SNP markers at the end of chromosomes and at the “border” between ends and middle of chromosomes. These were used to discriminate CO positions at ends and at the middle of chromosomes.

Table S3. t-Test of a small sample to estimate a putative correlation between the major BSN locus on chromosome 1 and average CO at the middle of chromosomes. Five individuals of the mapping population with the var. *bisporus* locus for BSN (*b*) and 5 individuals with the var. *burnettii* locus (*t*) for BSN were crossed with H39 (var. *bisporus*) resulting in hybrids with a BSN genotype of *b/b* or *b/t*. The t-Stat critical value is smaller than the t critical indicating that the 0 hypothesis (*b/b* = *b/t*) cannot be rejected. Although a not very convincing, a choice was made to select a majority of homokaryons from the mapping population with a var. *burnettii* genotype for the major BSN locus to increase the number of homokaryons in the F2 offspring and, therefore, reduce the labor costs.


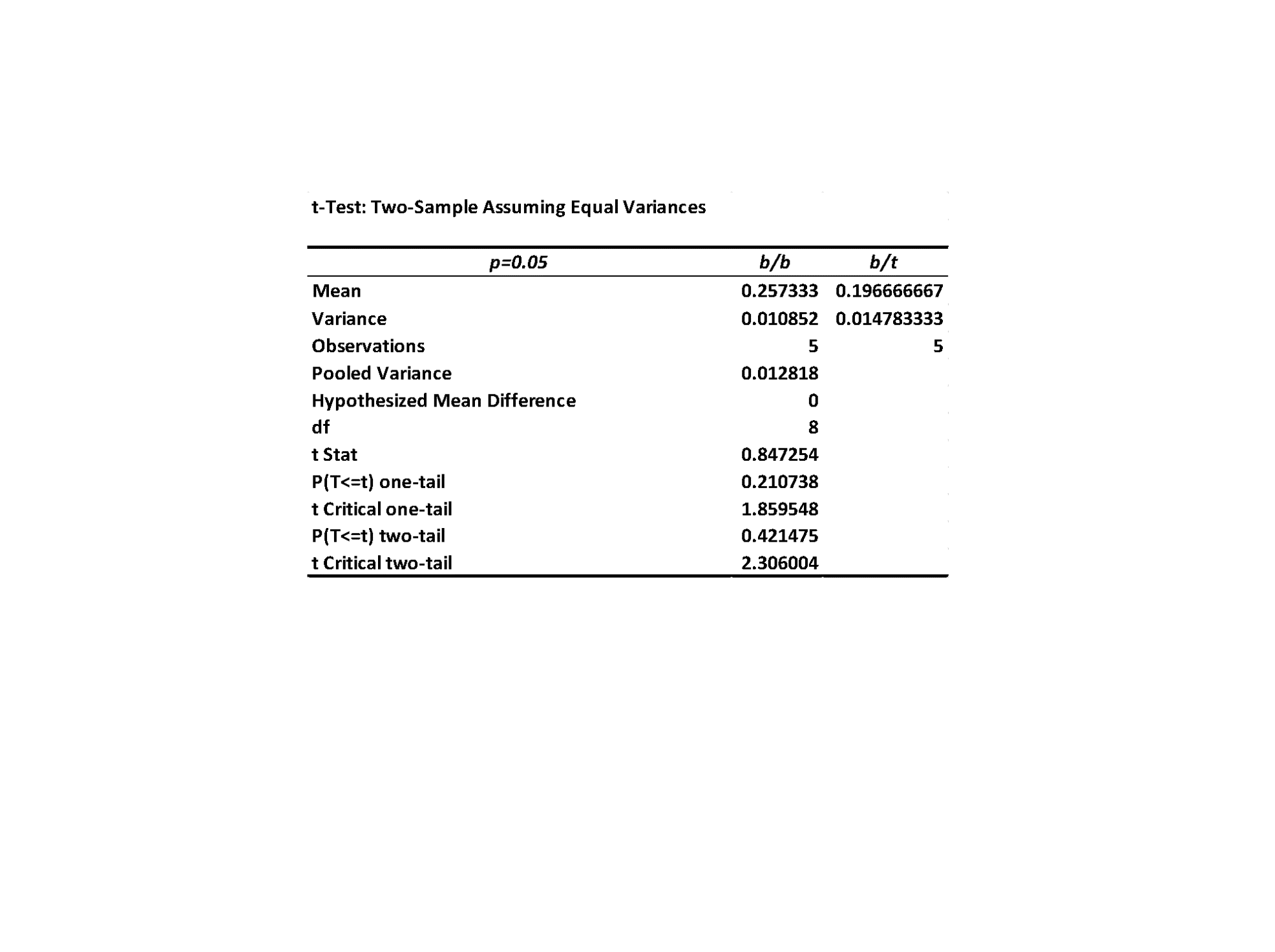

Supplement: Supplementary file 1 [file Data_Sheet_1.docx]
